# Supplementary material for: HIVprotI: an integrated web based platform for prediction and design of HIV proteins inhibitors
Source: J Cheminform. 2018 Mar 9;10:12. doi: 10.1186/s13321-018-0266-y (PMC5845081; doi:10.1186/s13321-018-0266-y)
Supplement: Supplementary file 2 — Additional file 2. Source code of HIVProtI web server. [file 13321_2018_266_MOESM2_ESM.zip › HIVprotI_Source-code/hivprotI/jsme/JSME_atom_highlight_demo.html]

# JSME atom highlight

|  |
| --- |
| 7 Highlight atom |
| Atom highlighted by mouse over: |
 Atom colors: 7,3,8,3,9,3,10,3,11,3,12,3,13,3 Set atom background color Reset Atom colors |

### Atom background color

There are 6 predefined pastel colors available: numbered 1 to 6. The color specification is character string containing a comma separated list of atom number and color index.  
Example: 1,2 : color atom #1 with color # 2  
Example: 1,2,10,4 : color atom #1 with color # 2 and color atom #10 with color #4  

### Bond background color

API not implemented yet.

## API

Note: All atom and molecule indices start at 1.

**public void setAtomToHighLight(int molIndex, int atomIndex)**  
The hightlight is temporary.

**public void setNotifyAtomHighLightChangeJSfunction(String notifyAtomHighLightJSfunction)**  
Receive a notification when JSME detects a mouse over one atom. The argument is the name of a JavaScript function that receives two arguments: the molecule index and the atom index.  
To cancel, set the argument to null.

**public void setAtomBackgroundColors(int molIndex, String atomAndColorCSV)**   
atomAndColorCSV must be an integer between 0 and 6. 0 means no background color.

**public void resetAtomColors(int molIndex)**
